# Long Non Coding RNA Based Regulation of Cerebrovascular Endothelium

**DOI:** 10.3389/fgene.2022.834367

**Published:** 2022-04-13

**Authors:** Samatha Mathew, Sridhar Sivasubbu

**Affiliations:** ^1^ CSIR Institute of Genomics and Integrative Biology (CSIR-IGIB), New Delhi, India; ^2^ Academy of Scientific and Innovative Research (AcSIR), Ghaziabad, India

**Keywords:** long non coding RNAs, blood brain barrier, neurovascular unit, cerebrovascular endothelial cells, organoids, animal models, lncRNA therapeutics

## Abstract

The rapid and high throughput discovery of long non coding RNAs (lncRNAs) has far outstripped the functional annotation of these novel transcripts in their respective cellular contexts. The cells of the blood brain barrier (BBB), especially the cerebrovascular endothelial cells (CVECs), are strictly regulated to maintain a controlled state of homeostasis for undisrupted brain function. Several key pathways are understood in CVEC function that lead to the development and maintenance of their barrier properties, the dysregulation of which leads to BBB breakdown and neuronal injury. Endothelial lncRNAs have been discovered and functionally validated in the past decade, spanning a wide variety of regulatory mechanisms in health and disease. We summarize here the lncRNA-mediated regulation of established pathways that maintain or disrupt the barrier property of CVECs, including in conditions such as ischemic stroke and glioma. These lncRNAs namely regulate the tight junction assembly/disassembly, angiogenesis, autophagy, apoptosis, and so on. The identification of these lncRNAs suggests a less understood mechanistic layer, calling for further studies in appropriate models of the blood brain barrier to shed light on the lncRNA-mediated regulation of CVEC function. Finally, we gather various approaches for validating lncRNAs in BBB function in human organoids and animal models and discuss the therapeutic potential of CVEC lncRNAs along with the current limitations.

## Graphical Abstract

Long non coding RNAs regulate the cerebrovascular endothelial processes.
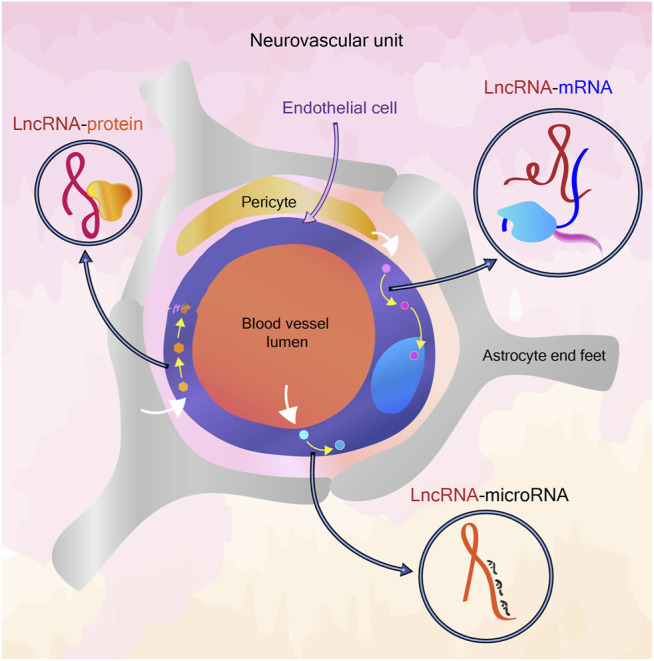

## Introduction

Endothelial cells (ECs) lining the blood vessel walls display functional, structural and biochemical heterogeneity across the body, depending on the cellular environment and blood flow ((Aird, 2007a), (Aird, 2007b)). Using endothelial cell-specific translating ribosome affinity purification, a recent study cataloged the *in vivo* translatomes of ECs of mice, showing the endothelial heterogeneity across vascular beds (Cleuren et al., 2019). Spatial transcriptomics of the brain in a cerebral cavernous malformation (CCM) mouse model could identify the differential involvement of arterial ECs versus venous ECs in the disease (Orsenigo et al., 2020). Endothelial cells also exhibit considerable plasticity in response to a variety of endothelial permeability modulators (Claesson-Welsh et al., 2021). Moreover, tissue-specific phenotype of the endothelium is not only decided by the environmental milieu, but is encoded by epigenetic marks as well (Aird, 2007a). The discovery of non-coding RNA (ncRNA) mediated regulation of epigenetic modifications and other cellular processes provide new avenues to explore the mechanisms of heterogeneity and plasticity of endothelial cells. With the availability of single cell endothelial transcriptomic data (Khan et al., 2019), there are many prospects ahead to decipher the complex network of the noncoding RNA and protein coding factor interplay in endothelial cells.

## Cerebrovascular Endothelial Cells

The heightened sensitivity of the brain tissue and neuronal signaling to pathogens, toxins and ionic imbalance, coupled with the inability to rapidly regenerate post damage has led to the evolution of a highly selective blood brain barrier (BBB) between blood and the brain parenchyma (Strange, 1992). Cerebrovascular endothelial cells (CVECs) are non-fenestrated, with exceptions in certain areas of the brain, and undergo very low rates of transcytosis allowing only lipid soluble molecules to pass through, sieving the movement of water-soluble solutes. CVECs are marked by abundant tight junctions at endothelial cell-cell boundaries, and express a large number of efflux proteins to exclude toxins and fewer leukocyte adhesion molecules to prevent inflammation. Additionally, they contain higher numbers of mitochondria (Obermeier et al., 2013; Daneman and Prat, 2015).

The cellular anatomy of the blood brain barrier consists of a conjugated system of endothelial cells, mural cells and glial cells, along with the basement lamina and glycocalyx. The signals sent by supporting cells to the endothelial cells determine the composition and permeability of the blood brain barrier. The signaling of the support cells are also influenced by the triggers from neurons (Obermeier et al., 2013). This neurovascular unit (NVU) is maintained throughout the life of a vertebrate for the normal functioning of the brain. The cerebrovascular endothelial cells rapidly respond to cues from the NVU, from both the luminal and abluminal sides. Apart from the regulation of angiogenesis and permeability during development by neurons, in adults, neuronal activity can send signals that affect the BBB permeability during stresses such as chronic sleep deprivation (Sweeney et al., 2019). In addition, in neuroinflammatory conditions such as ischemic stroke and psychiatric disorders, several cues lead to alteration in BBB permeability (Yang et al., 2019; Kealy et al., 2020).

## Interplay of Molecular Pathways in CVEC Function

During development, the Vascular Endothelial Growth Factor (VEGF) pathway is central to guidance and migration of endothelial cells into the neural tissue, during which time they possess tight junctions and transporter proteins, however with high levels of transcytosis and expression of leukocyte adhesion molecules (Bauer et al., 2014). Wnt/beta-catenin signaling pathway is specifically activated in the central nervous system (CNS), and regulates expression of nutrient transport molecules. The sonic hedgehog pathway further plays a role in the maintenance of the barrier properties (Obermeier et al., 2013). Signals from the pericytes and astrocytes and basement membrane also influence the maintenance of BBB (Daneman et al., 2010; Obermeier et al., 2013). The different cellular pathways involved in BBB establishment and permeability have been summarized recently (Tjakra et al., 2019).

## The Unknowns of BBB: Can Long Non Coding RNA Answer?

An elegant set of questions on the dynamicity of BBB have been raised by the Daneman group recently (Profaci et al., 2020). It is well understood that the BBB is not uniform in different parts of the brain and that the “breakdown” of BBB is also a non-uniform process. The non-coding RNA mediated regulation of the NVU has been known for more than a decade (Wang S.-W. et al., 2018). LncRNAs can exert a regulatory role at epigenetic, post-transcriptional, translational and post-translational levels (Xie and Wei, 2021), (Huang et al., 2021) while some lncRNAs are also known to function by coding for short peptides (Choi et al., 2019), (Hartford and Lal, 2020). LncRNAs are in particular known to be highly cell-type specific, unlike mRNA (Quinn and Chang, 2016), which could act as a potential factor in the variability of endothelial cells in general and variability within brain endothelial cells depending on brain region, age and disease conditions. Also, the abundance of a lncRNA can influence its functional role, which can add another tier of fine-tuning for cell-type specific properties (Grammatikakis and Lal, 2021). The tissue-specific transcriptomic and epigenomic signatures that differentiate CNS endothelial cells and other endothelial cells have been explored earlier (Sabbagh et al., 2018). The availability of transcriptomic data from the NVU at single cell resolution further opens up avenues to uncover novel regulators of cell function, including the less abundantly expressed lncRNAs (Kiss et al., 2020), (Mäe et al., 2021). In this review, we particularly focus on functionally validated lncRNAs associated with cerebrovascular endothelial cell pathways.

## Long Non Coding RNAs in Endothelial Function

Of the thousands of RNAs in a eukaryotic cell that defy the central dogma of biology, long non coding RNAs (lncRNAs) form a considerable proportion. NONCODEv5 has put together nearly 550 thousand lncRNA transcripts from 17 species, of which 172 thousand are of human origin (Fang et al., 2018). The ANGIOGENES database has catalogued protein coding RNAs and noncoding RNAs in endothelial cells from human, and the model organisms, mouse and zebrafish (Müller et al., 2016). Endothelial lncRNAs are known in development, function and vascular disease (Kok and Baker, 2019), (Jaé and Dimmeler, 2020). A recent review has classified vascular-associated lncRNAs based on their mechanism of action (Ono et al., 2021). Here we introduce some recent studies to spotlight the diverse regulation of endothelial function by lncRNAs:

The lncRNA *PUNISHER* or *AGAP2-AS1* is enriched in small extracellular vesicles (sEV) released by endothelial cells in the plasma of coronary artery disease (CAD) patients. *PUNISHER* interacts with *Heterogeneous Nuclear Ribonucleoprotein K* (*hnRNPK*), an RNA binding protein, and subsequently regulates the levels of VEGFA and endothelial cell proliferation in sEV recipient endothelial cells (Hosen et al., 2021). Another recent report shows lncRNA *NORAD* has a detrimental effect in CAD models by facilitating endothelial cell injury. *NORAD* is a nuclear lncRNA known to have multiple binding partners earlier, but in the said study was found to bind to HDAC6, through the RNA binding protein FUS. The *NORAD-*FUS-HDAC6 complex leads to H3K9 deacetylation at *VEGF* promoter and suppresses its transcription. The effects of *Norad* on endothelial cell injury were also demonstrated in atherosclerotic mice (Kai et al., 2021). In an oxidized LDL (oxLDL)-induced aortic endothelial cell model of atherosclerosis, the knockdown of lncRNA *AK087124* inhibited apoptosis and inflammation. *AK087124* acts as a sponge for *miR-224-5p*, which targets PTEN, a pro-apoptotic factor in the atherosclerotic model of aortic endothelial cells (Zhai et al., 2021).

LncRNA *VEAL2* was shown to interact with the kinase Protein kinase C beta 2 (PRKCB2) and prevent hyper-activation of the kinase by the small molecule diacylglycerol (DAG). This interplay regulates the turnover of junctional proteins VE-cadherin and beta-catenin, altering the permeability and angiogenic properties of human umbilical vein endothelial cells (HUVECs). *VEAL2* was found to be upregulated in the blood of diabetic retinopathy patients, and overexpression of the lncRNA in hyperglycemia model of HUVECs could attenuate PRKCB2-mediated hyper-permeability. A zebrafish knockout model for the ortholog *veal2* caused cranial hemorrhage, which could be rescued by the PRKCB2 inhibitor Enzastaurin (Sehgal et al., 2021). Aging related lncRNA *AERRIE* is important to maintain endothelial function and angiogenesis *in vitro*. Silencing of *AERRIE* led to DNA damage response mediated by YBX1. The study showed further that depletion of *AERRIE* induced DNA damage, and the damage response was activated by YBX1 as a consequence. Rescue with *AERRIE* could improve DNA repair in a doxorubicin mediated DNA damage model of endothelial spheroids (Pham et al., 2020). Endothelial-cell enriched lncRNA *LINC00961* encodes a micropeptide Small regulatory polypeptide of amino acid response (SPAAR). Knockdown of *LINC00961* in HUVECs prevents cell proliferation and angiogenesis. The lncRNA alone prevents angiogenesis while SPAAR induces vessel network formation and increases permeability, indicating that both the lncRNA and micropeptide have distinct functional roles, with independent protein partners (Spencer et al., 2020).

## The Evolving Roles of lncRNA in BBB Function

A plethora of noncoding RNAs are known to govern the BBB function and dysfunction, including small, long and circular non coding RNAs (Wang S.-W. et al., 2018), (Liu et al., 2021), (Gan et al., 2020). LncRNAs in BBB dysfunction have been extensively reported, with a view of identification of potential biomarkers. Here we attempt to collate the mechanistic role of lncRNA expressed in cerebrovascular endothelial cells and how they elicit regulation in known pathways.

### CVE LncRNAs Involved in Tight Junction Dynamics

Tight junctions (TJs) allow CVECs to form a size and charge selective barrier and many membrane complexes are assembled around TJs, helping in cell signaling and polarization of the endothelial cells. Three types of transmembrane proteins comprise TJs: Claudins, MARVEL-motif containing proteins such as occludin and immunoglobulin super family membrane proteins such as junctional adhesion molecule (JAM) isoforms. BBB usually contains claudin-3, 5 or 12, claudin-5 being the most abundant. Claudins interact with zona occludens (ZO) proteins via their C- terminals and their turnover is determined by phosphorylation marks. Zona occludens 1, 2 and 3 act as scaffold proteins and are instrumental in the formation of TJs, with ZO-1 and ZO-2 present in endothelial cells. Occludin interacts with the ZO proteins and cytoskeleton. Phosphorylation mediated marking of degradation of occludin regulates the permeability of the barrier. JAM B and C are known to be endothelial specific and loss of JAM-C causes cerebral hemorrhage. JAMs also mediate the transcellular migration of leukocytes in endothelial cells. The downregulation of TJ proteins have been reported in BBB disruption (Bauer et al., 2014). Several lncRNAs have been reported to regulate TJ protein levels and can affect barrier permeability and trans-endothelial electrical resistance (TEER).

*Lnc00462717* regulates levels of occludin in a blood-tumor barrier (BTB) model of brain microvascular endothelial cells (BMECs) by binding to polypyrimidine tract binding protein (PTBP1) and downregulating *miR-186-5p*. *miR-186-5p* directly binds to occludin 3′UTR and regulates its expression levels, thus altering the endothelial permeability (Zhang C. et al., 2020). LncRNA *HOTAIR* interacts with transcription factor upstream stimulatory factor 1 (USF1) and regulates levels of ZO-1, claudin-5 and occludin. *miR-148b-3p* binds to *HOTAIR* and USF1 transcripts and modulates the levels of the TJ proteins (Sa et al., 2017).

*Linc00174* titers the expression levels of the FOS Like 2, AP-1 Transcription Factor (FOSL2), which can regulate expressions of ZO-1, occludin and claudin-5. MicroRNAs *miR-138-5p* and *miR-150-5p* bind to both *Linc00174* and FOSL2 transcripts and bring down their levels, increasing permeability in the BTB model. It was seen that FOSL2 also has a feedback loop with *Linc00174* and binds to its promoter (Guo et al., 2019). *NEAT1* is a miRNA sponge of *miR-181d-5p* which regulates levels of RY-Box Transcription Factor 5 (SOX5) by binding to its 3’ UTR. SOX5 modulates levels of the tight junction proteins ZO-1, occludin and claudin-5 and thus *NEAT1* regulates BTB permeability (Guo et al., 2017).

LncRNA *MIAT* acts as a competing endogenous RNA (ceRNA) for miRNA *miR-140-3p*. ZO-1-associated kinase (ZAK) which phosphorylates NFκB-p65 is a target of *miR-140-3p*. NFκB-p65 is a transcription suppressor for the TJ proteins ZO-1, occludin, and claudin-5 (He et al., 2020). *LINC00094* sponges *miR-224-5p/miR-497-5p* in an Alzheimer’s disease (AD) microenvironment BBB model. *miR-224-5p/miR-497-5p* regulates Endophilin-1 post-translationally, which is a known factor in modulation of BBB permeability by altering expressions of ZO-1, claudin-5 and occludin through EGFR-ERK1/2 pathway (Zhu et al., 2019). LncRNA *XIST* is downregulated on ischemic insult while it is upregulated in the post-ischemic phase in patients and in a mouse model of cerebral ischemic stroke. In oxygen-glucose deprivation and restoration (OGD/R) model of ischemia in the murine brain endothelial cells, bEnd.3 cells, similar results were observed. Downregulation of *XIST* caused reduced expressions of integrin-α5, Kruppel-like transcription factor 4 (KLF4), claudin-5 and ZO-1 in ischemic conditions/OGD/R. Additionally, knockdown of *Xist* in bEnd.3 cells enhanced NF-κB activation and stalled angiogenesis. *Xist* acts as a sponge for *miR-92a*, which in turn regulates levels of integrin-α5 and KLF4. KLF4 overexpression can reduce levels of E-selectin, VCAM-1, ICAM-1 and p- NFκB (Wang C. et al., 2021).

### CVE lncRNAs Involved in VEGFA Signaling and Hypoxic Response

Signaling of the angiogenic Vascular Endothelial Growth Factor-A (VEGFA) is highly polarized in the cerebral vasculature, through the differential expression of the receptors VEGFR1 and VEGFR2 on the luminal and abluminal cell surfaces, respectively. In many scenarios, VEGFA signaling can induce disruption of the BBB by junctional turnovers, also altering TEER. Of note, VEGFA ligand binding on the abluminal side leads to leakage, while VEGFA binding on the luminal side has a protective role. VEGFR1 on the luminal side induces Akt pathway, and has a cytoprotective role by preventing apoptosis (Gerber et al., 1998), while VEGFR2 on the abluminal side activates p38 and increases barrier permeability (Dragoni and Turowski, 2018). VEGFA downregulates expressions of occludin and claudin-5, leading to the disassembly of TJs and increase in paracellular permeability of BBB (Argaw et al., 2009). VEGFA is also known to cause BBB breakdown through the eNOS pathway during CNS inflammation in a multiple sclerosis (MS) mouse model (Argaw et al., 2012). During hypoxic conditions, VEGF is shown to activate MMP-9, disrupting BBB (Valable et al., 2005). Pericytes and astrocytes release VEGF during hypoxic conditions of ischemia and stroke, leading to BBB disruption (Bai et al., 2015), (Li Y.-N. et al., 2014). Many lncRNAs are known to regulate VEGFA expression and thus affect angiogenesis and blood brain barrier permeability.

One of the earliest lncRNAs reported to have a function in cerebrovascular angiogenesis was *Meg3*, through the classical reverse genetic technique of generating a knockout. Whole gene knockout of lncRNA *Meg3* led to elevated levels of VEGF in brains of mice. Other VEGF pathway genes also showed differential expression in the brains of *Meg3*-null mice, which led to increase in microvessel density (Gordon et al., 2010). The overexpression of lncRNA *Anril* was shown to increase VEGF expression in the brains from Diabetes mellitus rat model with cerebral infarction. Apart from VEGF, the levels of NF-κB and p-IκB/IκB were also elevated in the brain tissues of this model, along with VEGFR1 and VEGFR2. This elevation in expression levels of genes of the VEGF pathway was then reflected as an increase in the microvessel density in the brain (Zhang B. et al., 2017).

LncRNA *MIAT* knockdown reduced levels of VEGF in HUVECs and reduced microvessel number and showed reduction in TJs in brains of an AD mouse model (Jiang et al., 2016). *MIAT* is a ceRNA sponge for *miR-150-5p*, which targets VEGF ((Yan B. et al., 2015). LncRNA *Meg8* is expressed in high levels in OGD challenged BMECs and its silencing inhibits proliferation and angiogenesis. Silencing of *Meg8* could be correlated with lower levels of VEGFA. *Meg8* acts as a sponge for *miR-130a-5p* which targets VEGFA. Injection of *Meg8* could diminish cerebral infarct volumes in middle cerebral artery occlusion (MCAO) model in rats (Sui et al., 2021).

LncRNA *Snhg1* which expresses abundantly in cerebral microvessels of ischemic mouse model and OGD induced BMECs is a ceRNA for *miR-18a*. Hypoxia-inducible factor 1-alpha (HIF1A) mediated activation of VEGF pathway is regulated by the *Snhg1/miR-18a* axis by the binding of *miR-18a* to suppress HIF1A expression and to determine the apoptosis and homeostasis of BMECs (Zhang L. et al., 2018). *Snhg1* is also known to act as a sponge for *miR-199a* in the OGD/R BMEC model, which can downregulate both HIF1A and VEGF and inhibit capillary formation by BMECs (Wang Z. et al., 2018). A third miRNA, *miR-338* is also sponged by lncRNA *Snhg1* in OGD-treated BMECs. *miR-338* binds to 3’ UTR of HIF1A and induces apoptotic pathways in the BMEC model of ischemia (Yang and Zi, 2019).

### CVE LncRNAs in PI3K-Akt Pathway

Depending on the factor that activates PI3K/Akt pathway, and the downstream effector of Akt, the outcomes of endothelial permeability can vary through assembly or disassembly of TJs (Cong and Kong, 2020). For instance, in ischemic hypoxia in rat neonates, the hematopoietic factor Granulocyte stimulating factor (G-CSF) activates PI3K/Akt/GSK-3β axis to increase BBB barrier properties and is anti-inflammatory. The authors of the study note that the protective effects of PI3K/Akt pathway were shown previously, which is mediated by reducing levels of VCAM-1, ICAM-1 and beta catenin, while upregulating claudin 3 and 5 (Li et al., 2015). On the contrary, in a rat model of brain injury and a parallel *in vitro* hypoxic model, PI3K/Akt/GSK-3β axis activation led to apoptosis of brain endothelial cells (Chen et al., 2017). Certain lncRNAs elicit the PI3K/Akt pathway to exert their regulatory action on CVECs.

LncRNA *MALAT1*, a ceRNA for *miR-126* in the OGD model of ischemia in human (HBMECs), has a positive effect on angiogenesis and cell viability via regulating PI3K-Akt pathway activation. *MALAT1* leads to endothelial cell apoptosis and inhibition of PI3K-Akt pathway in OGD HBMECs by sponging *miR-126* (Zhang L. et al., 2020). LncRNA *FAL1* has a protective effect against oxidative stress in OGD/R challenged HBVMECs. *FAL1* overexpression inhibits expressions of interleukin-6 (IL-6), monocyte chemotactic protein-1 (MCP-1) and high mobility group box-1 (HMGB1). Further, *FAL1* overexpression restored lowered phosphorylation levels of p21 (RAC1) activated kinase 1 (PAK1) and Akt in the OGD/R cells, and increased expression of proliferating cell nuclear antigen (PCNA), a downstream target. Thus, *FAL1* overexpression reversed cell injury induced by OGD/R (Gao M. et al., 2020).

### CVE lncRNAs in STAT3 Activation

Sphingosine-1-phosphate (S1P) pathway is activated in OGD/R and MCAO models of cerebral ischemia. Signal Transducer And Activator Of Transcription 3 (STAT3) gets activated downstream of S1P pathway and its inhibition protects BBB from OGD/R induced dysfunction (Nakagawa and Aruga, 2020). JAK/STAT3 pathway induction and microRNA mediated regulation of S1P receptor was also shown to cause BBB damage in rat model of septic encephalopathy (Chen S.-L. et al., 2020). Following are two examples of CVE lncRNAs with the potential to activate STAT3.

The abundant expression of lncRNA *Snhg3* in the BMVEC model of intracerebral hemorrhage (ICH) led to inhibition of proliferation and migration, apoptosis and loss of barrier properties. *Snhg3* overexpression increased levels of Tumor necrosis factor-like weak inducer of apoptosis (TWEAK) mRNA and protein and its receptor fibroblast growth factor-inducible 14 (Fn14). This then activated the STAT3 pathway by phosphorylation of STAT3 protein. STAT3 pathway activation led to the secretion of MMP-3 and MMP-9, increasing permeability. Combined inhibition of TWEAK, Fn14 and STAT3 could reverse effects of high expression of *Snhg3* in ICH BMVECs (Zhang J. et al., 2019). LncRNA *Malat1* knockdown reversed the upregulation of 15-lipoxygenase 1 (15-LOX1), pSTAT3 and VEGF in OGD/R BMECs. This suggested that *Malat1* induces angiogenesis through activation of STAT3 pathway in OGD/R BMECs (Wang C. et al., 2019).

### CVE LncRNAs in Autophagy

The process of autophagy is a systematic house-keeping process to remove damaged organelles or misfolded proteins by lysosomal degradation and in case of irreparable damage, the self-destruction of cells containing those (Mathiassen et al., 2017). Occludin degradation and subsequent increase in permeability has been shown to be induced via autophagy in OGD/R treated bEnd.3 cells and MCAO rat models (Kim et al., 2020). Autophagy mediated degradation of ZO-1 is known in high glucose conditioned BMECs in the OGD/R model (Zhang S. et al., 2018). Alternatively, protective effect of autophagy in OGD/R BMVECs by redistribution of ZO-1 on the membrane has also been shown (Li H. et al., 2014). In short term hypoxia injury, autophagy protects cerebrovascular endothelial cells from increased permeability and loss of TEER. Effect of autophagy in endothelial damage during ischemic stroke has been reviewed earlier (Kim et al., 2018). A couple of lncRNAs are known to modulate CVEC function through the regulation of the autophagy pathway.

In the OGD/R model of BMECs, *Malat1* sponges *miR-26b* which in turn regulates Unc-51 Like Autophagy Activating Kinase 2 (ULK2). Knockdown of ULK2 prevents autophagy in OGD/R BMECs. Hence *Malat1* acts as a protective agent in OGD/R by activating autophagy (Li Z. et al., 2017). Additionally in OGD induced bEnd.3 cells, *Malat1* is shown to act as a sponge for *miR-200c-3p* which binds to Sirtuin 1 (SIRT1) mRNA. Knockdown of SIRT1 inhibited LCB3II and increased p62 expression and increased cell death in bEnd.3 OGD/R cells. By maintaining the levels of SIRT1, *Malat1* protects bEnd.3 cells from cell death by inducing autophagy (Wang S. et al., 2019). LncRNA *PVT1* negatively regulates *miR-186* in glioma endothelial cells. *PVT1* overexpression induces formation of autophagosomes in the BTB model. *miR-186* binds to 3’ UTRs of Autophagy related 7 (Atg7) and Beclin1, preventing autophagy, while *PVT1* reverses this effect and facilitates endothelial cell autophagy in the BTB model (Ma et al., 2017a).

### CVE LncRNAs in Apoptosis

Once the vessels migrate into the nascent neural tissue, and the formation of the neural tissue is complete, the associated endothelial cells become quiescent. Apoptosis plays a major role in homeostasis of quiescent BMECs by removal of the dysfunctional cells. Apoptotic signals also directly regulate angiogenesis, and are particularly important during adult neurogenesis coupled with angiogenesis (Pozhilenkova et al., 2017). In disease conditions like stroke, apoptosis of BMECs leads to neuronal injury via increased BBB permeability and leads to vascular edema. Apoptosis of CVECs can occur through both intrinsic and extrinsic pathways of apoptosis, owing to various stimuli such as infections, amyloid proteins, inflammation, ischemia, and so on. Studies have shown that elevated levels of p53 and Bax, lower levels of Bcl-2, and activation of caspase-3 bring about apoptosis in BMECs. The ERK, JNK and p38-MAPK pathways are implicated in apoptosis induction in BMECs (Rizzo and Leaver, 2010). Several lncRNAs are known to perform anti-apoptotic or pro-apoptotic roles to determine outcomes of cerebrovascular endothelial cells in disease conditions.

*Malat1* is upregulated in the OGD model of BMECs and cerebral microvessels of ischemic mice after reperfusion. Knockout of *Malat1* amplifies ischemic injury in mice model. *Malat1* has an anti-apoptotic role in cerebral microvasculature by regulating the expression of the pro-apoptotic factor Bim. *Malat1* knockdown also increased levels of inflammatory cytokines like E-Selectin, MCP1 and IL-6. Further, *Malat1* was shown to directly interact with both Bim and E-selectin proteins (Zhang X. et al., 2017). LncRNA *SNHG16* acts as a sponge for *miR-15a-5p* in OGD/R HBMECs. *miR-15a-5p* binds to and targets Bcl-2 post transcriptionally. Hence *SNHG16* protects HBMECs from OGD/R apoptosis (Teng et al., 2020). *LncOGD-1006* is upregulated in OGD BMECs and is protective against apoptosis. Overexpression of *LncOGD-1006* increased expression of Bcl-2 and BCL2 associated agonist of cell death (BAD) proteins and downregulated cleaved caspase-3. *miR-184-5p* is sponged by *LncOGD-1006* which regulates expression of Caspase Activity And Apoptosis Inhibitor 1 (CAAP1). CAAP1 knockdown reversed effects of *LncOGD-1006* overexpression (Chen JY. et al., 2020). LncRNA *Neat1* was upregulated in OGD BMECs and is a sponge for *miR-377*. Knockdown of *Neat1* leads to apoptosis in OGD BMECs, increasing BAX expression and downregulating VEGF, SIRT1 and Bcl-xl. *miR-377* was shown to bind to 3′UTRs of VEGF, SIRT1 and Bcl-xl and hence *Neat1* acts anti-apoptotic by sponging *miR-377* (Zhou et al., 2019).

LncRNA *Meg3* knockdown protects rat BVMECs (RBMVECs) from OGD/R mediated apoptosis. *Meg3* directly interacts with p53 and modulates its downstream targets. The study showed that the p53 target NADPH Oxidase 4 (NOX4) expression is elevated in OGD/R RBVMECs and is regulated by *Meg3* via p53. NOX4 regulates levels of HIF1A and VEGF. Thus knockdown of *Meg3* acts anti-apoptotic and angiogenic in OGD/R RBVMECs (Zhan et al., 2017). LncRNA *FENDRR* induces apoptosis in hypertensive intracerebral hemorrhage (HIHC) model of HBMECs treated with thrombin. *FENDRR* acts as a sponge for *miR-126*, which can regulate VEGF levels. The study showed that VEGF downregulation could prevent apoptosis of thrombin-treated HBMECs (Dong et al., 2018).

LncRNA *Rmst* is abundantly expressed in OGD bEnd.3 cells and has a positive correlation with apoptosis while acting as a sponge for *miR-150* (Qiao et al., 2020). Another study showed *RMST* as a ceRNA for *miR-204-5p* in OGD models for promoting apoptosis. *miR-204-5p* was shown to target VCAM1 which induced permeability and apoptosis in CVECs (Yin et al., 2021). Overexpression of lncRNA *MIAT* induced apoptosis in intracranial aneurysm (IA) model of HBEC-5i cells by upregulating cleaved caspase-3, cleaved PARP1 and Bax, while downregulating Bcl-2. *MIAT* directly interacts with the MYC proto-oncogene and modulates the expression of the downstream gene Ectodermal-Neural Cortex 1 (ENC1). Silencing *Miat* and ENC1 independently could prevent apoptosis of cerebrovascular endothelial cells and protect against IA in a rat model (Li et al., 2020).

**FIGURE 1:**
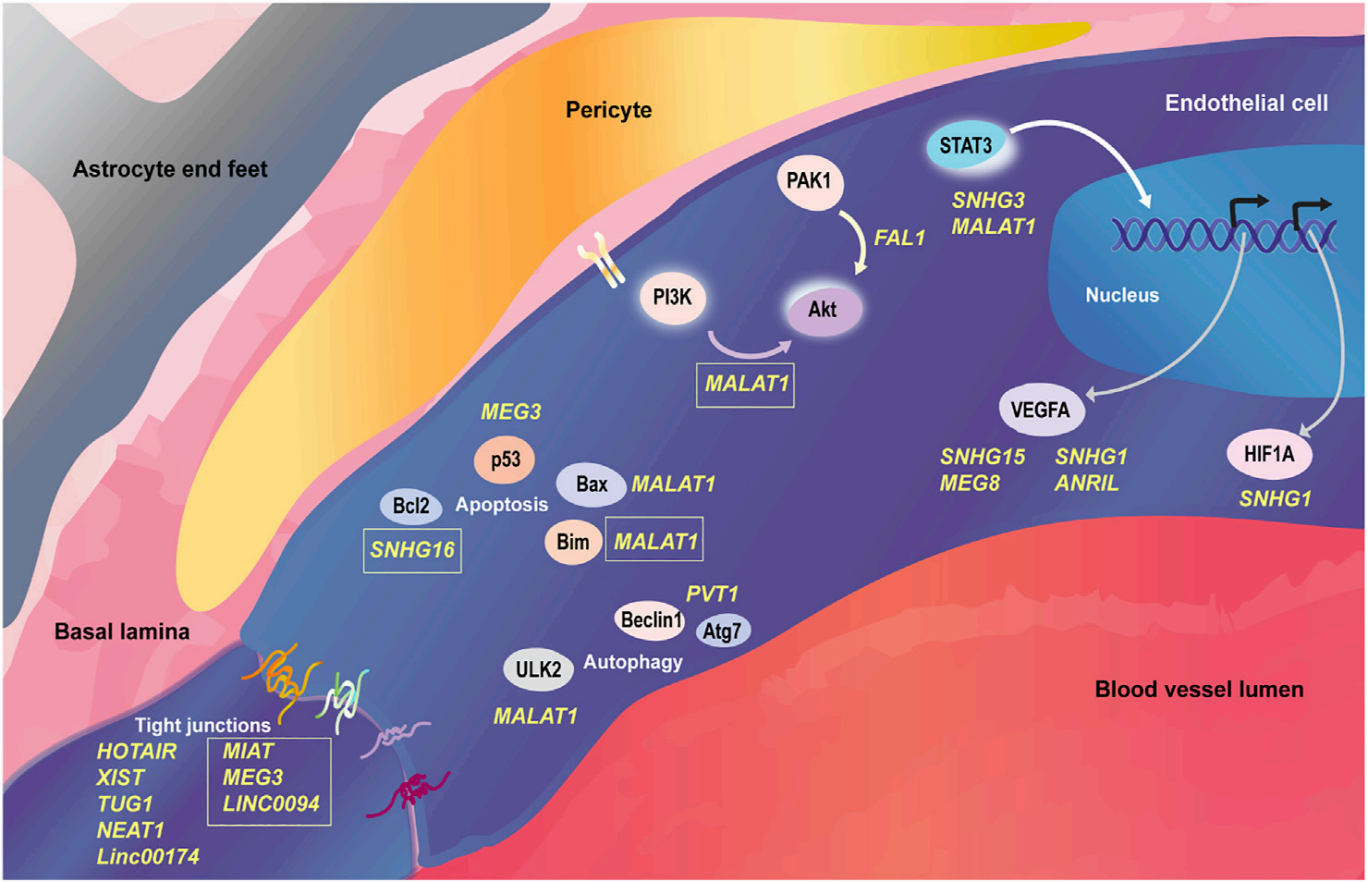
LncRNAs regulating cellular processes in cerebrovascular endothelial cells. The cerebrovascular endothelial cells of the neurovascular unit are governed by signaling pathways that respond to distinct cues from luminal and abluminal sides. Several lncRNAs have been discovered to regulate the protein coding gene players in cerebrovascular endothelial cells at post-transcriptional and post-translational levels within the signaling involving tight junction turn over, VEGF pathway, PI3K-Akt pathway, STAT3 activation, autophagy and apoptosis. LncRNAs are mentioned in yellow font in the figure; the ones in boxes negatively regulate their cognate pathways, while the rest have a positive role in their cognate pathways. All the references to the mentioned lncRNAs can be found in Table 1.

Apart from the afore-mentioned pathways, several other cerebrovascular endothelial lncRNAs have been reported to date. The entire list of lncRNAs reviewed here can be found in Table 1. We attempt to capture a snapshot of the lncRNA mediated regulation of CVEC pathways in Figure 1.

**TABLE 1:** List of lncRNAs expressed in cerebrovascular endothelial cells and their functional roles.

LncRNA Name	Associated Pathway in Cerebrovas-cular endothelial Cells	Interacting Partner	Effect on BBB	Mechanism	Disease/Condition	Model	References
*Lnc00462717*	Tight junction turn over	PTBP1	+	Regulation of occludin levels	Glioma	Co-culture BTB model	Zhang et al. (2020a)
*HOTAIR*	Tight junction turn over	miR-148b-3p	+	Modulation of ZO-1, claudin-5 and occludin levels	Glioma	Co-culture BTB model	Sa et al. (2017)
*XIST*	Tight junction turn over	miR-137	+	Modulation of levels of ZO-2 and CXCR7	Glioma	Co-culture BTB model	Yu et al. (2017)
*TUG1*	Tight junction turn over	miR-144	+	Modulation of ZO-1, claudin-5 and occludin levels	Glioma	Co-culture BTB model	Cai et al. (2015)
*MALAT1*	Tight junction turn over	miR-140	+	Modulation of ZO-1, claudin-5 and occludin levels	Glioma	Co-culture BTB model	Ma et al. (2016)
*Linc00174*	Tight junction turn over	miR-138-5p and miR-150-5p	+	Modulation of ZO-1, claudin-5 and occludin levels	Glioma	Co-culture BTB model	Guo et al. (2019)
*NEAT1*	Tight junction turn over	miR-181daysay-5p	+	Modulation of ZO-1, claudin-5 and occludin levels	Glioma	Co-culture BTB model	Guo et al. (2017)
*MEG3*	Tight junction turn over	miR-330-5p	-	Modulation of ZO-1, claudin-5 and occludin levels	Glioma	Co-culture BTB model	Shen et al. (2018)
*MIAT*	Tight junction turn over	miR-140-3p	-	Modulation of ZO-1, claudin-5 and occludin levels	Glioma	Co-culture BTB model	He et al. (2020)
*LINC00094*	Tight junction turn over	miR-224-5p/miR-497-5p	-	Modulation of ZO-1 and occludin levels	Alzheimer’s disease microenvironment	Co-culture BTB model	Zhu et al. (2019)
*LncRSPH9-4*	Tight junction turn over	miR-17-5p	-	Modulation of ZO-1 and occludin levels	Meningitic *E. coli* Infection	Human BMECs	Xu et al. (2021a)
*Xist*	Tight junction turn over	miR-92a	+	Modulation of ZO-1 and claudin-5 occludin levels	Cerebral ischemia	OGD/R murine brain endothelial cells and bEnd.3 cells	Wang et al. (2021a)
*Miat*	VEGF pathway, Tight junction turn over	None	+	Modulation of ZO-1 and occludin levels	Alzheimer’s disease	Mice	Jiang et al. (2016)
*Meg3*	VEGF pathway	None	*	Modulation of VEGF levels in brain	MEG3 knockout	MEG3 null mice	Gordon et al. (2010)
*Anril*	VEGF pathway	None	*	Modulation of VEGF levels in brain	Diabetes mellitus with cerebral infarction	Rats	Zhang et al. (2017a)
*MALAT1*	VEGF pathway	miR-205-5p	*	Modulation of VEGF levels	Cerebral ischemia	OGD/R HBMEC	Gao et al. (2020a)
*Snhg12*	VEGF pathway	miR-150	*	Modulation of VEGF levels	Cerebral ischemia	OGD bEnd.3 cells, MCAO mice	Zhao et al. (2018)
*SNHG15*	VEGF pathway	miR-153	*	Modulation of VEGFA levels	Glioma	Glioma-conditioned hCMECs	Ma et al. (2017b)
*Meg8*	VEGF pathway	miR-130a-5p	*	Modulation of VEGFA levels	Cerebral ischemia	OGD bEnd.3 cells, MCAO rats	Sui et al. (2021)
*Snhg1*	Hypoxia pathway	miR-18a	*	Modulation of HIF1A levels	Cerebral ischemia	OGD mice BMECs, MCAO mice	Zhang et al. (2018a)
*Snhg1*	VEGF-Hypoxia pathway	miR-199a	*	Modulation of VEGF and HIF1A levels	Cerebral ischemia	OGD/R model of mice BMECs	Wang et al. (2018b)
*Snhg1*	Hypoxia pathway	miR-338	*	Modulation of HIF1A levels	Cerebral ischemia	OGD mice BMECs	Yang and Zi, (2019)
*MALAT1*	PI3K-Akt pathway	miR-126	*	Activation of PI3K and Akt	Cerebral ischemia	OGD human BMECs	Zhang et al. (2020b)
*FAL1*	PAK1-Akt pathway	None	*	Activation of PAK1 and Akt	Cerebral ischemia	OGD/R human BMECs	Gao et al. (2020c)
*Snhg3*	STAT3 pathway	None	+	Activation of STAT3	Intracerebral hemorrhage	ICH rats, OGD plus themin rat BMVECs	Zhang et al. (2019a)
*Malat1*	STAT3 pathway	none	*	Activation of 15-LOX1/STAT3 pathway	Cerebral ischemia	OGD/R mice BMECs, MCAO mice	Wang et al. (2019a)
*Malat1*	Autophagy	miR-26b	*	Modulation of ULK2 levels	Cerebral ischemia	OGD/R mice BMECs	Li et al. (2017b)
*Malat1*	Autophagy	miR-200c-3p	*	Modulation of SIRT1 levels	Cerebral ischemia	OGD bEnd.3 cells	Wang et al. (2019b)
*PVT1*	Autophagy	miR-186	*	Modulation of Atg7 and Beclin1 levels	Glioma	Glioma-conditioned human CMECs	Ma et al. (2017a)
*Malat1*	Apoptosis	Bim and E-selectin	*	Modulation of Bim levels and pro-inflammatory cytokines	Cerebral ischemia	OGD mice BMECs, MCAO mice	Zhang et al. (2017b)
*Malat1*	VEGF pathway, Apoptosis	miR-143	*	Modulation of VEGF, ET-1, vWF, and MMP-9 levels	Intracranial aneurysm	IA rats, Vascular endothelial cells from IA rat tissue	Gao et al. (2020b)
*MALAT1*	Apoptosis	none	*	Modulation of MDM2, p53 and BAX levels	Cerebral ischemia	OGD/R human BMECs, MCAO mice	Zhang et al. (2018c)
*SNHG16*	Apoptosis	miR-15a-5p	*	Modulation of Bcl-2 levels	Cerebral ischemia	OGD/R human BMECs	Teng et al. (2020)
*LncOGD-1006*	Apoptosis	miR-184-5p	*	Modulation of CAAP1 levels	Cerebral ischemia	OGD bEnd.3 cells	Chen et al. (2020a)
*Neat1*	Apoptosis, VEGF pathway	miR-377	*	Modulation of SIRT1, VEGFA, and BCL-XL levels	Cerebral ischemia	OGD mice BMECs	Zhou et al. (2019)
*Meg3*	Apoptosis, VEGF pathway, Hypoxia pathway	p53	*	Modulation of p53 and NOX4 levels	Cerebral ischemia	OGD/R rat BMVECs	Zhan et al. (2017)
*FENDRR*	Apoptosis, VEGF pathway	miR-126	*	Modulation of VEGFA levels	Hypertensive intracerebral hemorrhage	Human BMECs treated with thrombin	Dong et al. (2018)
*Gas5*	Apoptosis	miR-34b-3p	*	Modulation of EPHA4 levels	Cerebral ischemia	OGD/R bEnd.3 cells	Shen et al. (2021)
*Snhg1*	Apoptosis	miR-298	*	Modulation of SIK1 levels	Cerebral ischemia	OGD/R bEnd.3 cells	Zhou et al. (2021)
*Rmst*	Apoptosis	miR-150	*	Modulation of MMP2 and MMP9 levels, change in caspase-3 activity	Cerebral ischemia	OGD bEnd.3 cells	Qiao et al. (2020)
*RMST*	Apoptosis	miR-204-5p	*	Modulation of VCAM1 levels	Cerebral ischemia	OGD human BMECs and bEnd.3 cells	Yin et al. (2021)
*MIAT*	Apoptosis	MYC	*	Modulation of ENC1 levels	Intracranial aneurysm	Vascular endothelial cells HBEC-5i, IA rats	Li et al. (2020)
*Meg3*	Ferroptosis	None	*	Modulation of p53 and GPX4 levels	Cerebral ischemia with hyperglycemia	OGD rat BMVECs with hyperglycemia	Chen et al. (2021b)
*H19*	Ferroptosis	miR-106b-5p	*	Modulation of ACSL4 levels	Intracerebral hemorrhage	ICH human BMVECs	Chen et al. (2021a)
*LINC00346*	Angiogenesis	ANKHD1	*	Modulation of EGFL7 and ROBO4 levels	Glioma	Glioma-conditioned human CMECs	Yang et al. (2020)
*Dancr*	Angiogenesis	miR-33a-5p	*	Modulation of XBP1 levels	Cerebral ischemia	OGD rat BMECs	Zhang et al. (2020c)
*Miat*	Angiogenesis	miR-204-5p	-	Modulation of HMGB1 levels	Cerebral ischemia	OGD rat CMECs, MCAO rats	Deng et al. (2020)

BTB, blood tumor barrier; BMECs/BMVECs, brain microvascular endothelial cells; OGD/R, oxygen-glucose deprivation and restoration; OGD, oxygen-glucose deprivation; MCAO, middle cerebral artery occlusion; CMECs, cerebral microvascular endothelial cells; IA, intracranial aneurysm. Effect on BBB is represented as + (maintains the BBB),−(induces BBB permeability), * (no information in the study).

## Studying lncRNAs in Different BBB Models

This review has compiled studies on lncRNAs in BBB cell culture models and animal models, mostly in various disease contexts. However, it is widely accepted that the cell culture models of cerebrovascular endothelial cell monolayers or even co-cultures with glial/mural cells are inadequate in representing the blood brain barrier, which is closely regulated by other components of the neurovascular unit. The 2-D and 3-D *in vitro* models of BBB and *in vivo* animal models such as rodent and zebrafish models of BBB and their applicability were summarized recently (Bhalerao et al., 2020), (Jackson et al., 2019). Of particular interest in *in vitro* models is human induced pluripotent stem cell (iPSC)-derived brain microvascular endothelial cell model and multi-cellular organoids which incorporate various cell types of the NVU (Logan et al., 2019), (Lacalle-Aurioles et al., 2020). An intriguing humanized model which involves introduction of human BBB organoids into murine systems has also been explored (Waldau, 2019).

Near identical brain microvasculature across species corresponding to the number of neurons gives a hint that non-human models are good candidates to study BBB function (Wong et al., 2013). Rodent models such as mice and rats are popular models owing to BBB architecture similar to humans, and their evaluation can be done post-mortem or through live techniques such as PET, MRI or two-photon imaging (Jackson et al., 2019). Tissue clearing techniques such as CLARITY and PACT have additionally opened up the possibility of visualizing fixed but intact rodent brain (Tomer et al., 2014), (Woo et al., 2016). The well-annotated invertebrate model *Drosophila*, has been used to understand BBB dysfunction and drug delivery despite the limited similarity with mammalian system of having only a glial barrier (Cuddapah et al., 2019), (Schirmeier and Klämbt, 2015). In zebrafish, the blood brain barrier genesis occurs between 3 and 10 days post fertilization and this gives an opportunity to study the progressive selective permeability over time using dye diffusion based assays. Adult zebrafish BBB is known to be highly similar to human BBB (Li Y. et al., 2017). Zebrafish has also been shown to be an amicable model to understand the role of pericytes and smooth muscle cells in BBB development and maintenance (Bahrami and Childs, 2018). Similarly, the utility of transparent *Xenopus* tadpoles to study blood brain permeability has also been proven (De Jesús Andino et al., 2016). Despite the availability of a range of animal models to study BBB, it is known that the human BBB has unique biochemical signatures which cannot be recapitulated completely (Wong et al., 2013). Hence a combined approach of using animal models and human BBB organoids may be a rational approach to study BBB phenomena.

## Future Perspectives for Studying lncRNA in BBB Function

The advancements in deep sequencing, with the latest introduction of single-cell sequencing have led to the recording of thousands of novel lncRNAs. The rapidly developing bioinformatic frameworks have also allowed the prediction of biological interactions and functions of lncRNAs. These ambiguous molecules however are experimentally validated in disproportionately lower numbers and this acts as a bottle-neck in understanding their physiologically relevant roles. The past decades have seen the use of genome-editing tools such as TALENs and CRISPR-Cas9 systems to probe functions of novel genes. CRISPR based systems for RNA interference, activation and gene editing have already been tested for high throughput functional screening of lncRNA in erstwhile contexts (Esposito et al., 2019). Further, innovative systems to circumvent effects of conventional CRISPR-Cas9 approaches, including epigenetic silencing and post-transcriptional editing can also help study lncRNA function (Awwad, 2019), (Nuñez et al., 2021). In recent times RNA-editing versions of Cas protein have also emerged, which could be used as a novel strategy (Xu et al., 2020).

A majority of lncRNA functional studies are carried out in cell lines which are amenable to relatively less cumbersome methods of screening. However, as discussed in the previous section, the complex neurovascular unit and the blood brain barrier property as its function is captured limitedly by cell culture based models. Three-dimensional hydrogel based organoids are being explored for modeling the NVU (Potjewyd et al., 2021). The 3D structure will allow emulating the mechanical and biochemical properties of the neurovascular milieu. A recent study successfully developed a vascularized brain organoid model in mice with necessary BBB properties (Cakir et al., 2019). Another recent study has shown the editing using CRISPR-Cas9 systems in BBB organoids to identify receptor mediated transcytosis in brain endothelial cells (Simonneau et al., 2021). Therefore in order to study functions of novel lncRNA, BBB organoids can be leveraged by creating gene edited models to understand the role of lncRNA in BBB establishment and maintenance. Further such organoid models can be used for drug screening to identify small molecules that can modulate BBB permeability and function (Bergmann et al., 2018).

In lieu of the poor sequence conservation of lncRNAs, identification of lncRNAs in animal models can illuminate hitherto unknown regulatory angles in well studied pathways. Identification of functionally conserved human lncRNA has been exemplified in the case of lncRNAs *jpx* (mouse) (Karner et al., 2020) and *veal2* (zebrafish) (Sehgal et al., 2021) even in the absence of sequence or structural conservation, albeit with a conserved interacting protein partner. This provides a framework to look beyond sequence-based conservation for identifying lncRNA candidates that regulate well-conserved pathways. Rodent models, which are the most popular models to study BBB physiology have been used to study endothelial and BBB-related lncRNA (Cleuren et al., 2019), (Orsenigo et al., 2020). The very recent cell-type atlas release by the BRAIN Initiative Cell Census Network (BRAIN Initiative Cell Census Network, 2021) that captures nuclear transcriptomic signatures, along with epigenetic state information from mouse motor cortex, adds another powerful dataset for understanding novel transcripts including lncRNA in mammalian brain (BRAIN Initiative Cell Census Network (BICCN), 2021). Zebrafish can be deployed for large functional screens, using anti-sense oligos like morpholinos and CRISPR-based systems (Gut et al., 2017), (Ranjan et al., 2021). Endothelial lncRNA datasets from zebrafish have been reported by several groups (Sehgal et al., 2021), (Müller et al., 2016), while an entire compendium of zebrafish conserved lncRNA is available on the ZFLNC database (Hu et al., 2018). A transcriptome wide sgRNA-guide design may be attempted in zebrafish, coupled with *in vivo* imaging to track modulations in BBB integrity. The CRISPR toolkit has been proven to be efficient in *Xenopus* and *Drosophila* as well, and may well be exploited for understanding lncRNA function in BBB (Bhattacharya et al., 2015), (Port et al., 2020). The drug-based alterations in lncRNA levels in different model systems (Jiang et al., 2019) can further have potential applications in deriving drug-targets among lncRNA identified from BBB.

**FIGURE 2:**
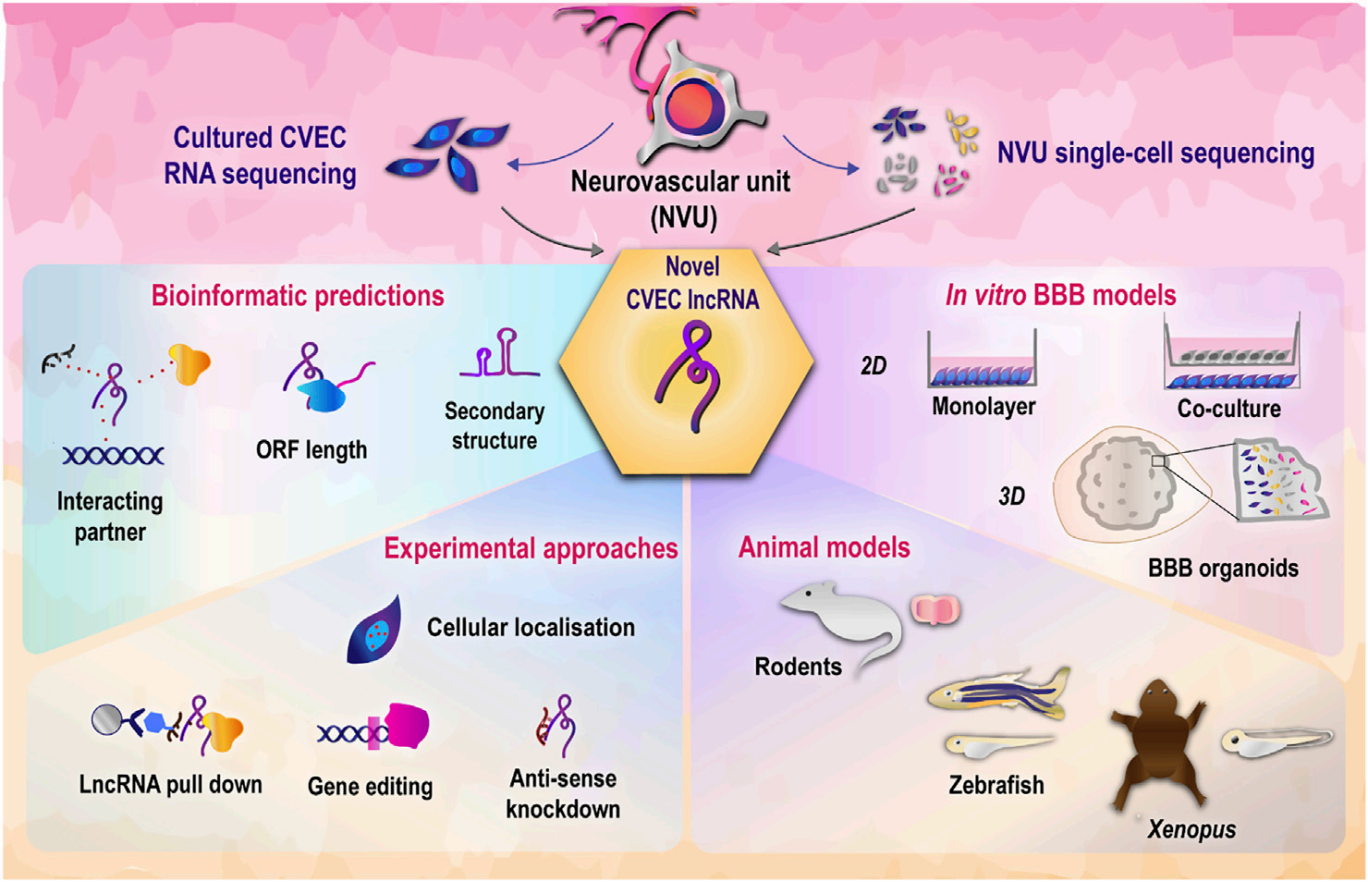
Strategies to decipher functional roles of cerebrovascular endothelial lncRNAs. LncRNAs discovered from cerebrovascular endothelial cells through transcriptomic studies can be functionally validated using a combination of bioinformatics and experimental approaches (Jalali et al., 2015), (McDonel and Guttman, 2019). *In vitro* and *in vivo* model systems employed in tandem will allow in deciphering the physiological roles of lncRNA in cerebrovascular endothelial function (Jackson et al., 2019). CVEC, cerebrovascular endothelial cells; BBB, blood brain barrier.

It can be seen from Table 1 that currently redundant roles have been assigned to a handful of lncRNAs, leaving open the door for functionally validating novel and unannotated candidates. Of note, many of the referred studies have observed that the lncRNA function is exclusive to the respective disease model and does not hold true in the context of homeostasis. Hence, orthogonal validations are needed to strengthen the understanding of lncRNA-mediated regulation of CVEC function. A recent study invalidated several ncRNA studies, underscoring the need to thoroughly test predicted interactions and mechanisms of ncRNA (Mitschka and Mayr, 2021). Stringent and parallel functional validation approaches will allow in confidently placing the lncRNA-mediated regulatory mechanisms in the bigger picture of cerebrovascular endothelial function. We provide a glimpse of the different strategies and models that can be employed for studying lncRNA in cerebrovascular endothelial function in Figure 2.

## Therapeutic Potential of CVEC lncRNA

LncRNAs are expected to be effective biomarkers and drug targets, due to their low and spatio-temporally restricted expression patterns. This section discusses the expression patterns and levels of lncRNAs and how these correlate with function and ultimately the therapeutic applicability of lncRNA candidates. We further attempt to understand how lncRNAs can be potential therapeutic targets in the context of CVECs.

### Expression Patterns of lncRNAs

From 24 human tissues and cell types, Cabili et al. showed that long intergenic RNA (lincRNA), a subset of lncRNA, have highly tissue-specific expression patterns, with the majority having low expression levels. In particular, the brain and testes expressed lncRNAs that are highly tissue-specific. The study reported 4,200 plus lincRNA, and concluded that 78% of lincRNA are tissue-specific, compared to 19% of protein coding genes, after normalizing for expression levels (Cabili et al., 2011). Based on transcriptomic data of 15 human cell lines, compared to protein coding genes, lncRNAs show greater unique expression in cell types (Djebali et al., 2012). The study showed that 10% lncRNA transcripts were found in all cell lines, compared to 29% found in only a single cell line. The corresponding numbers for protein coding transcripts were 53 and 7%. The lower and tissue restricted expression of lncRNAs was replicated during the ENCODE phase 2 project using 16 human tissue samples from RNA-seq data. About 11% of lncRNAs were found in all tissue types, with 65% being the corresponding proportion for protein coding transcripts. The study cautions that the tissue-restricted expression may be a false read out owing to the lower expression levels of lncRNAs. The study also used custom microarrays for five human cell lines, 17 tissues and nine region-wise brain samples-all representing a total of 31 cell types-which confirmed lower expression of lncRNAs with respect to protein coding transcripts (Derrien et al., 2012). Further an expression analysis of lncRNAs from The Cancer Genome Atlas (TCGA) including 5,037 samples corresponding to 13 cancer types showed cancer type-specific differential expression of lncRNA (Yan X. et al., 2015). In summary, the understanding of the tissue-specific nature of lncRNAs could be dictated by technical limitations of the detection techniques, however even considering these limitations, the relative abundance is relevant to biological function of lncRNA (Grammatikakis and Lal, 2021). In particular, studies have shown on comparing bulk RNA-sequencing and single cell sequencing that certain lncRNAs are relatively abundantly expressed in a very specific subset of cells, but predictably may show a lower signal in the data from a pool of cells from a tissue (Yan et al., 2013), (Liu et al., 2016).

### LncRNA Expression and Apparent Functional Correlation

Depending on the type of function and mode of action, the cellular abundance of a lncRNA may vary. For instance, nuclear localized lncRNAs may be of low abundance if they are *cis*-acting or *trans*-acting with specific targets, while *trans*-acting lncRNAs with genome-wide roles or cytoplasmically localized lncRNA may have relatively higher copy numbers in a cell (∼1,000 or more copies per cell) (Grammatikakis and Lal, 2021). Chromatin associated lncRNAs are known to be expressed as low as ∼0.3 copies per cell. In comparison protein coding transcript copy numbers lie in the ranges of approximately 10–800 thousands. Thus, the copy number of lncRNA is directly dependent on the kind of function a lncRNA performs (Wu et al., 2021). The quantification of abundance of lncRNA at cellular and tissue-level contexts will be paramount in both determining the function and therapeutic targeting (Kopp and Mendell, 2018). Given that lncRNAs are an amorphous class of molecules with a wide range of functions and varying spatio-temporal abundance, the therapeutic targeting of individual lncRNAs may need customized approaches.

### Strategies for Therapeutic Applications of lncRNAs

Clinical trials around the therapeutic potential of lncRNAs have picked up in the past few years. Therapeutic targeting of small non coding RNA such as miRNA has been demonstrated earlier (Winkle et al., 2021), and is being explored in many disease contexts including cerebrovascular conditions such as stroke (Xu Y. et al., 2021). A search for the key word “long non coding RNA” retrieved 69 results on https://www.clinicaltrials.gov/(As on 27 February 2022). Most of these studies have assessed/intend to assess the potential of lncRNAs as biomarkers in various conditions. Several lncRNAs have already shown potential as biomarkers in cancers (Gutschner et al., 2018). Of note, lncRNAs such as *ANRIL* and *MIAT* were primarily identified as differentially expressed transcripts in cardiovascular diseases (Holdt et al., 2010), (Ishii et al., 2006). Hence the applicability of lncRNAs as biomarkers has been well documented and with the increasingly explored transcriptomic data, more and more lncRNA biomarkers may come to the fore.

For considering the potential of lncRNAs as druggable targets, various strategies have been adopted. It has been shown in a phase I clinical trial that lncRNAs of mitochondrial origin can be targeted successfully. FDA approved the use of single-stranded phosphorothioate anti-sense oligo (ASO) against anti-sense mitochondrial lncRNA for treatment of solid tumors in advanced metastatic cancer and the ASO was shown to be well tolerated (Chen Y. et al., 2021). A cell-type specific targeting of cancer cells with higher levels of EGFR was shown to be possible by coupling an anti-EGFR aptamer with anti-*HOTAIR* siRNA in a cell line model of triple-negative breast cancer (Wang YL. et al., 2021). The strategies to explore the therapeutic potential of lncRNAs have been reviewed earlier (Renganathan and Felley-Bosco, 2017), (Winkle et al., 2021), (Schwarzmueller et al., 2020). RNA interference, ASO based targeting and CRISPR based targeting are slated to be most effective for modulating levels of lncRNAs (Arun et al., 2018). ASOs have been shown to be effective in mouse disease models against lncRNAs *anti-GATA6* (Zhu et al., 2018) and *MALAT1* (Arun et al., 2016). Small molecule targeting has also been shown to have some application against lncRNAs such as *GAS5* and *MALAT1* (Winkle et al., 2021). This mode of targeting of lncRNAs can be facilitated by understanding the secondary and tertiary structures of lncRNAs and sterically blocking functional motifs (Arun et al., 2018). Combining different approaches of lncRNA targeting have also been attempted (Chen Y. et al., 2021). On the other end, RNA therapeutics where non coding RNAs are used to target another transcript has also been explored. RNA therapeutics can be delivered to the system of interest using carriers and adjuvants, or using viral vectors (Poller et al., 2018). In addition, exosome based and nano-particle based delivery approaches of RNA therapeutics including delivery of lncRNA in cardiovascular disease are also being tested. Exosome-based delivery can in particular help in cell-specific targeting of RNA therapeutics by incorporating molecules on the exosome membranes that can detect and bind to cell-type specific markers (Lu and Thum, 2019). Intravenous delivery of ncRNAs involved in cardiac functioning has been shown to successfully have a therapeutic effect in a mouse model of cardiovascular disease (Quattrocelli et al., 2013).

### Targeting CVEC lncRNAs for Therapeutic Applications

The therapeutic applicability of lncRNAs in cerebrovascular diseases has been revealed from transcriptomic analyses. In conditions such as ischemic stroke, differential expression of several lncRNAs could be seen (Deng et al., 2018), and the levels of *H19*, *ANRIL*, *NEAT1* and other candidate lncRNAs seem to correlate with the severity of stroke (Gan et al., 2020). These lncRNAs showed marked upregulation in ischemic stroke and are considered as biomarkers. Possibilities of targeting lncRNAs across cardiovascular diseases have been reviewed earlier (Gomes et al., 2017). Small molecule based targeting in cardiac arrhythmias have been shown to lead to lowering of the levels of lncRNAs exacerbating the disease (Zhang Y. et al., 2019).

As described before, BBB dysfunction can lead to neurological conditions and vice versa. It is therefore important to look at therapeutic interventions that can manage potential breach of the blood brain barrier. Some of the approaches to prevent excessive permeability of the BBB is by targeting the VEGF pathway players and the downstream matrix metalloproteases. Further the use of antago-miRs and small molecules that can elevate levels of junctional proteins of the endothelial cells have also been proven effective (Archie et al., 2021). Engineered Wnt ligands have also been shown to be effective in preventing deterioration of BBB properties in conditions such as glioblastoma and stroke (Martin et al., 2022). This review has described CVEC lncRNAs across different pathways including VEGF and Wnt-related pathways. Targeting of lncRNAs that are potential modulators of such pathways which have already been shown to have drug targets to modulate BBB function provides a framework to explore novel therapeutic mechanisms in BBB dysfunction.

Increasing number of transcriptomic studies, especially at single cell resolution, are identifying unique EC differentiating patterns providing opportunities to specifically target ECs subtypes such as CVECs. Single cell sequencing of cells from brain vasculature of mice could identify unique transcriptomic signatures in endothelial cells, microglia, oligodendrocytes and fibroblasts (Lin et al., 2021). Cerebrovascular endothelial cells themselves have been shown to be heterogeneous in a cerebral cavernous malformation mouse model (Orsenigo et al., 2020). Five distinct EC types were identified from single cell sequencing of glioblastoma (GBM) tumor vessels from human patients. The EC phenotypes showed varying breakdown extents of the blood brain barrier property. Additionally, the ECs isolated from GBM tissue showed different gene signatures than ECs isolated from peripheral brain tissue (Xie et al., 2021). While these studies did not explicitly shed light on the non-coding RNA players, it will be interesting to see future studies on the lncRNA markers across CVEC sub-populations. This can pave the way for cell type-specific targeting of CVEC lncRNA using approaches such as exosome-based delivery of ASOs or RNA therapeutics (Lu and Thum, 2019).

### Current Challenges in Therapeutic Targeting of lncRNAs

While low copy numbers of lncRNAs that can be targeted can be an advantage because they may need lower dosages of targeting agents, there are several challenges to be tackled. One of the primary considerations in lncRNA targeting is the appropriate classification of lncRNAs based on abundance, processing, function and structure for designing therapeutics to each class for better effectiveness (Poller et al., 2018). As mentioned earlier, signals from bulk RNA sequencing may give a misleading read out on the cell types from expressing lncRNA. With the widespread use of single cell sequencing, this limitation can be overcome in the near future. Alternatively, visualization techniques such as fluorescence *in situ* hybridization can hint at a cellular subset-specific origin of a lncRNA. Further, a known functional relevance of lncRNA and proven conserved function if discovered in a model organism can be factors imperative for selection of a lncRNA therapeutic target (Gutschner et al., 2018). An additional consideration for RNA therapeutics based targeting of lncRNAs is the immunogenicity and toxicity of such agents, apart from issues of stability, specificity and delivery (Winkle et al., 2021), (Chen Y. et al., 2021). Hence, testing the therapeutic potential of lncRNAs may need several layers of examination and clear understanding of the molecular mechanism across a variety of models and conditions.

## Conclusion

The homeostasis of cerebrovascular endothelial cells forming the blood brain barrier is under the complex regulation of multiple pathways, which are primarily dictated by surrounding cells and various local environmental cues. This allows CVECs to exhibit dynamicity and plasticity in response to cues in the neurovascular milieu. Many lncRNAs have been shown to perform a regulatory role in cellular processes of CVECs, suggesting the presence of an under-studied mechanistic layer added by lncRNAs in CVEC function. Adopting appropriate models mimicking the cerebrovascular endothelial cells, blood-brain barrier and neurovascular unit can be an important strategy to gain a true picture of the underlying mechanisms of lncRNAs. The past decades have seen lncRNA studies that have mostly focused on a small subset of highly and ubiquitously expressed lncRNAs that are technically easier to probe with the available strategies to understand lncRNA function. This review is a compendium of several such studies that have cataloged the functional mechanism of lncRNAs in CVECs that could have redundant or overlapping roles in other cell types as well. With an increase in the annotation of the thousands of novel lncRNAs identified from bulk RNA sequencing and cell-type specific sequencing, in the future CVEC specific lncRNA signature may emerge that can be specifically targeted in the context of maintaining and modulating the blood brain barrier.

## Glossary

15-LOX1: 15-lipoxygenase 1
AERRIE: Age and EndMT Regulated RNA In Endothelium
Akt: Protein kinase B
ANRIL: Antisense non-coding RNA in the INK4 locus
Atg7: Autophagy related 7
BAD: BCL2 associated agonist of cell death
BBB: Blood brain barrier
Bcl-2: B-cell lymphoma 2
BMECs/BMVECs: Brain microvascular endothelial cells
BTB: Blood tumor barrier
CAAP1: Caspase Activity and Apoptosis Inhibitor 1
ceRNA: competing endogenous RNA
CMECs: Cerebral microvascular endothelial cells
CRISPR: Clustered regularly interspaced short palindromic repeats
CVECs: Cerebrovascular endothelial cells
DAG: Diacyl glycerol
DANCR: Differentiation Antagonizing Non-Protein Coding RNA
EC: Endothelial cells
EGFR: Epidermal growth factor
ENC1: Ectodermal-Neural Cortex 1
eNOS: Endothelial nitric oxide synthase
ERK: Extracellular signal-regulated kinase
FAL1: Focally amplified lncRNA on chromosome 1
FENDRR: FOXF1 Adjacent Non-Coding Developmental Regulatory RNA
Fn14: Fibroblast growth factor-inducible 14
FOSL2: FOS Like 2, AP-1 Transcription Factor
FUS: Fused in sarcoma
GAS5: Growth Arrest Specific 5
G-CSF: Granulocyte stimulating factor
GSK-3β: Glycogen synthase kinase 3 beta
H19: H19 Imprinted Maternally Expressed Transcript
HBEC-5i: Human Brain Endothelial Cell line-5i
HDAC6: Histone deacetylase 6
HIF1A: Hypoxia-inducible factor 1-alpha
HIHC: Hypertensive intracerebral hemorrhage
HOTAIR: HOX antisense intergenic RNA
HUVECs: Human umbilical vein endothelial cells
IA: Intracranial aneurysm
ICAM-1: Intercellular adhesion molecule-1
ICH: Intracerebral hemorrhage
JAK: Janus kinase
JAM: Junctional adhesion molecule
KLF4: Kruppel-like transcription factor 4
LncRNA: long non coding RNA
MALAT1: Metastasis Associated Lung Adenocarcinoma Transcript 1
MAPK: Mitogen-activated protein kinase
MARVEL: MAL and related proteins for vesicle trafficking and membrane link
MCAO: Middle cerebral artery occlusion
MCP-1: Monocyte chemotactic protein-1
MEG3: Maternally expressed 3
MEG8: Maternally Expressed 8
MIAT: Myocardial infarction associated transcript
MMP: Matrix metalloproteinase
MRI: Magnetic resonance imaging
MYC: MYC Proto-Oncogene, BHLH Transcription Factor
NEAT1: Nuclear Enriched Abundant Transcript 1
NORAD: Non-Coding RNA Activated by DNA Damage
NOX4: NADPH Oxidase 4
NVU: Neurovascular unit
OGD: Oxygen-glucose deprivation
OGD/R: Oxygen-glucose deprivation and restoration
OxLDL: Oxidized LDL
PAK1: p21 (RAC1) activated kinase 1
PARP1: Poly [ADP-ribose] polymerase 1
PCNA: Proliferating cell nuclear antigen
PET: Positron emission tomography
PI3K: Phosphoinositide 3-kinase
PRKCB2: Protein kinase C beta 2
PTEN: Phosphatase and tensin homolog
PVT1: Plasmacytoma variant translocation 1
RMST: Rhabdomyosarcoma 2 associated transcript
S1P: Sphingosine-1-phosphate
SIRT1: Sirtuin 1
SNHG: Small Nucleolar RNA Host Gene
SOX5: RY-Box Transcription Factor 5
SPAAR: Small regulatory polypeptide of amino acid response
STAT3: Signal Transducer and Activator Of Transcription 3
TALEN: Transcription activator-like effector nucleases
TEER: Transendothelial electrical resistance
TWEAK: Tumor necrosis factor-like weak inducer of apoptosis
ULK2: Unc-51 Like Autophagy Activating Kinase 2
USF1: Upstream stimulatory factor 1
VCAM-1: Vascular cell adhesion protein 1
VEAL2: Vascular endothelial associated lncRNA-2
VEGF: Vascular endothelial growth factor
VEGFR: Vascular endothelial growth factor receptor
XIST: X-inactive specific transcript
YBX1: Y box binding protein 1
ZAK: ZO-1-associated kinase
ZO: Zona occludens
